# Orthodontics: Bracket Materials, Adhesives Systems, and Their Bond Strength

**DOI:** 10.1155/2016/1329814

**Published:** 2016-10-13

**Authors:** Andrea Scribante, Rosalia Contreras-Bulnes, Mona A. Montasser, Pekka K. Vallittu

**Affiliations:** ^1^Unit of Orthodontics and Paediatric Dentistry, Section of Dentistry, Department of Clinical, Surgical, Diagnostic and Paediatric Sciences, University of Pavia, Pavia, Italy; ^2^Facultad de Odontología, Universidad Autónoma del Estado de México, Toluca, MEX, Mexico; ^3^Orthodontic Department, Faculty of Dentistry, Mansoura University, Mansoura, Egypt; ^4^Institute of Dentistry, Faculty of Medicine, University of Turku, Turku, Finland

Adhesive interfaces influence greatly clinical success of modern dentistry. Durability of the interface can be determined by using several* in vitro* testing methods. Shear bond strength tests are widely used in dentistry and they are well suitable for testing orthodontic materials bonded to teeth. The first study that analyzed shear bond strength of orthodontic appliances appeared in international literature in the late 1970s [1]. Nowadays, more than one thousand reports have been conducted in order to analyze various factors influencing shear bond strength of orthodontic brackets. Precise interpretation of the shear bond strength test results should, however, take into account other types of stress which are occurring at the interface during testing.

Previous studies that evaluated bond strength analyzed different variables related to adhesive system (composite or resin-modified glass ionomer), bonding surface (enamel, ceramic, or metal), antibacterial agents (added to adhesive system), bracket material (steel, ceramic, or plastic), bracket type (conventional, self-ligating, or lingual), attachment base (with various mesh sizes and shapes), brace mesh or surface pretreatment (such as sandblasting) [2], bracket placement force, enamel conditioning (with etchants or lasers), enamel pretreatment (with protecting or bleaching agents), and enamel contaminants (such as blood or saliva). The effect of any of these factors may differ when rebonding orthodontic brackets [2–10]. Moreover, bonding studies have been applied to test not only orthodontic brackets but also other materials bonded to tooth structure during active or passive orthodontic treatment (such as customized CAD CAM bases, disinclusion buttons, and fiber reinforced composites bars and nets) [11].

During over 35 years of orthodontic bonding studies, a standardized technique has been reached, but many differences in methods among different studies still remain [12]. Due to increased ethical requirements, the human teeth used are usually wisdom teeth or first premolars (extracted for orthodontic reasons). Bovine teeth are collected in slaughterhouses in deciduous or permanent dentition. Tooth selection includes intact buccal enamel and no cracks due to extraction procedure. After extraction, teeth are stored in thymol, water, or artificial saliva, whereas formalin and alcohol are no more used in order to avoid adverse effects on bond strength measurement.

Brackets or jigs are bonded to teeth with an adhesive system and subsequently, or after artificial ageing specimens, are placed in a testing machine with the adhesion surface parallel to shearing force.

Predominantly, a shear force is applied with a steel tip with standardized crosshead speed until adhesive failure. Debonding force is recorded in newtons and then often converted into megapascals, which is the unit of stress at the interface. Special attention needs to be paid to ensure the geometry of the bonding site of the bracket allows calculation of stress. In the case of complex form of the bonding site, it is correct to report the bonding properties as debonding load. Moreover, enamel and appliance surfaces are analyzed under optic magnification and an Adhesive Remnant Index (ARI) is assigned to give information of the location of the adhesive failure [13]. ARI score is calculated evaluating the amount of adhesive left on tooth and appliance surfaces after debonding. ARI scale usually ranges from 0 to 3 (0: no resin remaining on tooth; 1: less than 50% resin remaining on tooth; 2: more than 50% resin remaining on tooth; 3: 100% resin remaining on tooth).

As it is a standard procedure in biomedical research, statistical analyses are performed with a high enough number of test specimens (i.e., teeth). Descriptive statistics (mean, standard deviation, minimum, median, and maximum values) are calculated for the groups which are compared. The normality of the data can be calculated (e.g., using the Kolmogorov-Smirnov test). Parametric (e.g., ANOVA) or nonparametric (e.g., Kruskal-Wallis) tests are then applied and parametric (e.g., Tukey) or nonparametric (e.g., Mann–Whitney) post hoc tests are used to show differences among various groups. On the other hand, for ARI scores a Chi Squared test is often applied. Significance for all statistical tests is almost always predetermined at* P* < 0.05.

In the literature, there are not clear guidelines about shear force limits, but in fact a good orthodontic biomaterial should allow good adhesion in order to sustain masticatory forces (with a minimum bond strength of 5–10 MPa) [14]. On the other hand, adhesion forces should not be too strong in order to avoid enamel loss after debonding (40–50 MPa) [15]. Therefore, the ideal orthodontic biomaterial should have bonding forces included in the interval of 5–50 MPa, even if these limits are mostly theoretical.

When considering ARI index, even if methods of measurement could influence score assignment results [16], ARI score is nowadays widely used in bonding studies to assess and discuss adhesive left on tooth surface after debonding. Generally, a score of “0” is often related to lower shear bond strength values and is often related to contaminants over enamel that can reduce bond strength. On the other hand, an ARI score of “3” means less risk of enamel fracture after bracket debonding but polishing procedures are longer as more adhesive remains on tooth surface [9]. Therefore, an orthodontic biomaterial should aspire to a mixed adhesion modality (ARI “1” and “2”).

In conclusion, bonding studies represent one of the first steps of materials testing and should be followed by* in vivo* clinical studies in order to confirm the* in vitro* results. Therefore, although some criticisms have been stated against bonding studies in orthodontics, bonding tests are still a valid instrument to test new brackets, adhesives, jigs, pad, and other biomaterials bonded to tooth surface.

On the basis of these considerations, the present special issue has been proposed to explore new variables of bonding studies. These new topics have been about the Er:YAG laser-recycled ceramic orthodontic brackets, the transmission of curing light through treated dental tissues, the effect of removal of enamel on rebonding strength of resin composite, the bond strength of different bonding systems on enamel and restorative materials, and the bonding of metal attachments to sandblasted porcelain and zirconia.

The Guest Editors do hope that the present special issue would be interesting for the readers of the journal and wish that the present work could encourage other researchers for future, original, interesting bond strength studies.

*Andrea Scribante* *Rosalia Contreras-Bulnes* *Mona A. Montasser* *Pekka K. Vallittu*
